# The Coupling Effect of O_2_ and H_2_S on the Corrosion of G20 Steel in a Simulating Environment of Flue Gas Injection in the Xinjiang Oil Field

**DOI:** 10.3390/ma11091635

**Published:** 2018-09-06

**Authors:** Xiankang Zhong, Yanran Wang, Jianjun Liang, Long Chen, Xiaoqin Song

**Affiliations:** 1 School of Oil and Natural Gas Engnieering, Southwest Petroleum University, Chengdu 610500, China; zhongxk@swpu.edu.cn (X.Z.); wyr201621000527@163.com (Y.W.); 2 Engineering Technology Institute, Xinjiang Oil Filed Company, CNPC, Karamay 834000, China; liangjj@petrochina.com.cn (J.L.); cyycl@petrochina.com.cn (L.C.)

**Keywords:** O_2_ Corrosion, H_2_S Corrosion, Combined Effect, Flue Gas Injection

## Abstract

Flue gas injection for heavy oil recovery has received a great deal of attention, because it is more cost effective than lots of other injection methods. However, the corrosion could occur easily, because the flue gas usually contains corrosive gases such as CO_2_, H_2_S, and O_2_. In this work, the corrosion behaviors of G20 steel in flue gas injection environment simulating Xinjiang oil field (China) were investigated using weight loss measurement and surface characterization techniques. The effect of environments including the O_2_-containing environment, the H_2_S-containing environment, and the O_2_-H_2_S-coexisting environment on the corrosion of G20 steel in gas phase and liquid phase was discussed. The results show that the corrosion rate of G20 steel in the O_2_-H_2_S-coexisting environment is much higher than the sum of corrosion rates of the O_2_-containing environment and the H_2_S-containing environment, regardless of the gas phase and the liquid phase. This indicates that there is a coupling effect between O_2_ and H_2_S, which can further accelerate the corrosion of steel in O_2_-H_2_S-coexisting environment. The results of surface characterization demonstrate that in a typical flue gas injection environment, the corrosion products are composed of FeCO_3_, FeS, FeO(OH), and elemental sulfur. Elemental sulfur could obviously accelerate the corrosion of steel. Therefore, it can be considered that the coupling effect of O_2_ and H_2_S on corrosion of G20 steel in flue gas injection environment is caused by the formation of elemental sulfur in corrosion products.

## 1. Introduction

Flue gas injection for heavy oil recovery has received a great deal of attention, because it is more cost effective than lots of other injection methods such as natural gas injection [1]. The flue gas as an injectant for enhanced oil recovery is usually composed of multiple components including N_2_, CO_2_, O_2_, H_2_S, hydrocarbons, and water vapor. It is well known that the presence of CO_2_, O_2_, and H_2_S in a wet environment may cause corrosion of metal and alloys. Therefore, there is high corrosion risk for the surface piping and subsurface tubulars in flue gas injection process. Usually, two main strategies are employed to minimize the potential of corrosion: (I) reduce the oxygen content to a minimum by conducting the combustion in a fuel-rich environment and (II) eliminate the possibility of vapour condensation by ensuring that the flue gas is always above its dew point [2]. However, the corrosion cannot still be completely avoided, especially during the practical production process that faces the complex environment.

According to the composition of flue gas mentioned above, in the presence of aqueous solution or water vapor, CO_2_, O_2_, and H_2_S would cause corrosion of metal materials including surface injection piping and subsurface tubulars. CO_2_ corrosion has been extensively investigated in the past decades [3,4,5,6,7,8]. Great progress has been made on CO_2_ mechanisms and CO_2_ corrosion prediction based on the dedicated efforts of researchers. However, the presence of O_2_ and H_2_S in CO_2_-containing environment makes the corrosion progress more complicated. 

In CO_2_-O_2_ corrosive environment, as a strong oxidant, O_2_ could not only directly participate the cathodic reduction reaction during the corrosion process but also change the formation of corrosion products. For example, Choi et al. investigated the O_2_ effect on the corrosion of carbon steel in supercritical CO_2_-water environments. It was found that the corrosion rate of carbon steel increased with the addition of O_2_ to the system due to the inhibition effect of O_2_ on the formation of protective FeCO_3_ [9]. Sun et al. also found that O_2_ could inhibit the formation of FeCO_3_ and dominate the corrosion process in the supercritical CO_2_-H_2_O-O_2_ system [10]. Lin et al. reported that in the CO_2_-O_2_ environment, dissolved O_2_ damaged the integrity of corrosion scale of 3Cr steel by precipitation of Fe(OH)_3_ at localized area, which caused the non-uniform distribution of Cr, leading to the pitting corrosion and high corrosion rate [11]. 

The presence of H_2_S in the CO_2_-containing environment can significantly change the corrosion progress and mechanism of metal and alloys. Abelev et al. studied the effect of H_2_S on Fe corrosion in CO_2_-saturated brine and found that small H_2_S concentrations (5 ppmv) showed an inhibition effect on corrosion and higher H_2_S (>50 ppmv); the corrosion rate increased rapidly, but it was still below the corrosion rate for the H_2_S-free solution [12]. Choi et al. found that the addition of low-level (100 ppmv) H_2_S into CO_2_ saturated 1 wt % NaCl at pH 3 and 4 induced a rapid decrease in the corrosion rate of 1018 carbon steel. This inhibition effect was attributed to the formation of thin FeS film on the steel surface that suppressed the anodic dissolution [13]. Sun et al. pointed out that a low concentration (2000 ppmv) of H_2_S accelerated the corrosion rate of X65 steel owing to the additional cathodic reaction and the water phase precipitation promoted by H_2_S in supercritical CO_2_-H_2_O-H_2_S system [10].

As mentioned above, the effect of a single component (either O_2_ or H_2_S) on the corrosion in the CO_2_-containing environment has been widely investigated. However, studies related to the corrosion in CO_2_-containing environment where O_2_ and H_2_S exist simultaneously are still limited [10,14,15,16]. To our best knowledge, the coupling effect of O_2_ and H_2_S in the simulated flue gas injection environment has been rarely reported.

Flue gas injection as an enhanced oil recovery technique has been put on the agenda in Xinjiang oil field (China), where the flue gas contains 15 mol % CO_2_, 1.5 mol % O_2_, 1.2 mol % CH_4_, 600 ppmv H_2_S, and about 82 mol % N_2_. The composition of flue gas indicates that it is highly corrosive for surface piping and subsurface tubulars if this gas is accompanied by the water vapour condensation. Therefore, the investigation on corrosion of metal materials in the simulating flue gas injection environment is highly significant for the assessment of safety risk prior to the implementation of this project. In this work, the coupling effect of O_2_ and H_2_S on the corrosion of surface piping (G20 steel) in the simulating flue gas injection environment in the Xinjiang oil field was investigated using weight loss measurement and surface characterization techniques.

## 2. Materials and Methods

### 2.1. Materials and Solution

**Figure 1:**
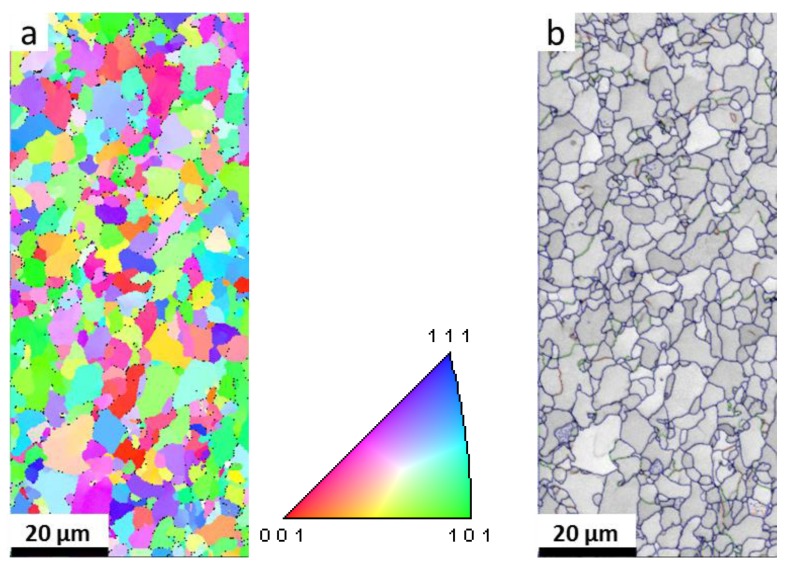
EBSD measurements of G20 steel: (**a**) inverse pole figure map; (**b**) grain boundary map.

The specimens used in this work were cut from a G20 steel pipe with the following chemical composition (wt %): C 0.18, Si 0.21, Mn 0.5, P 0.03, and S 0.03, with Fe making up the balance. The starting microstructure of G20 steel characterized using electron backscatter diffraction (EBSD, a Zeiss-Crossbeam XB 1540 FIB scanning electron microscope with a Hikari camara, EDAX, AMETEK, Inc. America) is shown in Figure 1. It is demonstrated that G20 steel mainly contains ferrite and cementite (Fe_3_C), and the grain size distribution is homogenous. The cementite mainly distributes at the grain boundary. The presence of ferrite and cementite can also be confirmed in the X-ray diffraction (XRD) results in Section 3.

The specimens used for the weight loss measurements and surface analysis were cuboids with dimension of 50 mm × 10 mm × 3 mm. Prior to the experiment, each specimen was grounded sequentially using up to 800 grit SiC paper, degreased in an ultrasonic bath with acetone for 10 min, rinsed in ethanol, and dried under the nitrogen flow. The weight of the specimen was taken using an analytical balance with the accuracy of 0.1 mg. Each experiment was performed by exposing five specimens to the simulated flue gas injection environment in autoclave. Three specimens were used for weight loss measurements; the fourth and the fifth were used for surface analysis.

Corrosive solution, which was made up from analytical grade reagents and deionized water, simulated the formation water in Xinjiang oil field; its ionic compositions are listed in Table 1. The total salinity was 9254 mg/L. The solution pH was 7.0. Prior to the experiment, the corrosive solution was deaerated by purging CO_2_ (99.99%) gas for 4 h.

### 2.2. Corrosion Experiment in Autoclave

**Figure 2:**
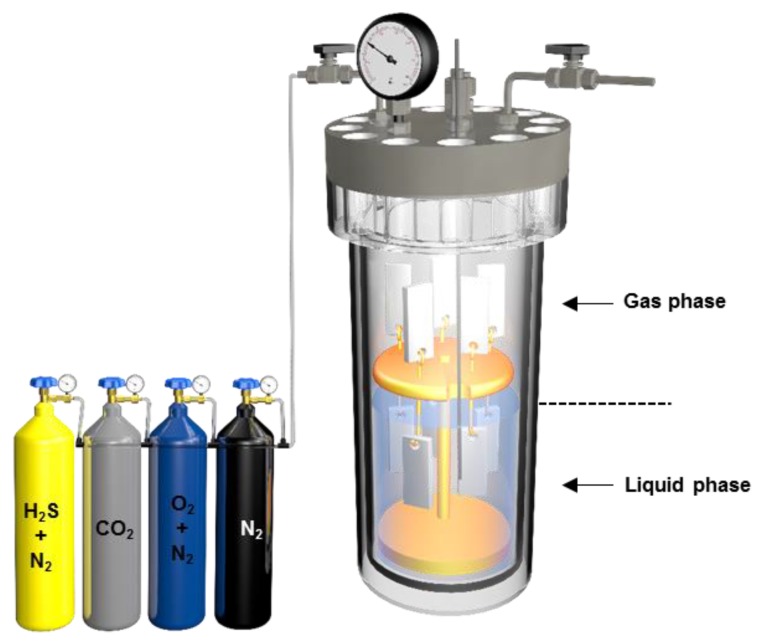
Schematic diagram of the setup for simulating the environment of flue gas injection in laboratory.

A 6 L Hastealloy C276 autoclave was used for the corrosion experiment in the present work. The schematic is shown in Figure 2. Two corrosive environments including gas phase and liquid phase were made simultaneously in the one autoclave. Gas phase environment is closer to environment of flue gas injection. As a comparison, the corrosion of G20 steel in liquid phase was also studied. Five specimens for weight loss measurements and surface analysis were mounted into the Teflon holder in the gas phase. Also, other five specimens were installed on the holder in the liquid phase, as is shown in Figure 2. Then, the autoclave was closed and vacuumed by a vacuum-pumping system. 2.5 L of deaerated solution was then introduced into the autoclave. After the autoclave was heated to 60 °C, CO_2_, O_2_, and H_2_S were injected into the autoclave to a desired partial pressure or concentration. Finally, a booster pump was used to add N_2_ into the autoclave to reach a pressure of 15 MPa. All experiments were conducted under static conditions. Table 2 lists the conditions and parameters of corrosion test. The experiment duration was 120 h. It should be noted that since the aim of this work is to study the interaction between H_2_S and CO_2_ during the corrosion of G20 steel, the CO_2_ corrosion experiment was not conducted specifically.

After the experiment, specimens for XRD and scanning electron spectroscopy (SEM, ZEISS EVO MA 15 SEM, Oberkochen, Germany) were rinsed with ethanol and dried in a vacuum oven until time came for analysis, while specimens that were used for the analysis of cross-section of corrosion products were rinsed with ethanol, dried, and immediately mounted into the epoxy resin. Specimens for weight loss measurement were brushed to mechanically remove loose corrosion products, rinsed with acetone, exposed to the Clarke solution (ASTM G103) to dissolve the remaining corrosion products [17,18], dried again in N_2_ gas flow, and finally weighed.

### 2.3. Evaluation of Corrosion Rates

The corrosion rate was calculated as follows:

(1) $$CR=87,600\frac{\Delta m}{\rho A\Delta t},$$

in which *CR* is the corrosion rate (mm/y), Δm presents the weight loss of the specimen before and after corrosion (g), ρ is density of specimen (g/cm^3^), A is area of specimen exposed to the corrosive solution (cm^2^), and t is duration of corrosion (h).

### 2.4. Characterization of Corrosion Products

The surface morphology and the cross-sectional morphology of corrosion product formed on specimen surfaces were examined using a ZEISS EVO MA 15 SEM. The elemental composition of the corrosion products was analyzed by EDS, which was coupled with the SEM. The crystal structure of the corrosion products was investigated by X-ray diffraction on X’pert PRO with a Co-K X-ray tube (PANalytical, Almelo, The Netherlands).

## 3. Results

### 3.1. Corrosion Rates

**Figure 3:**
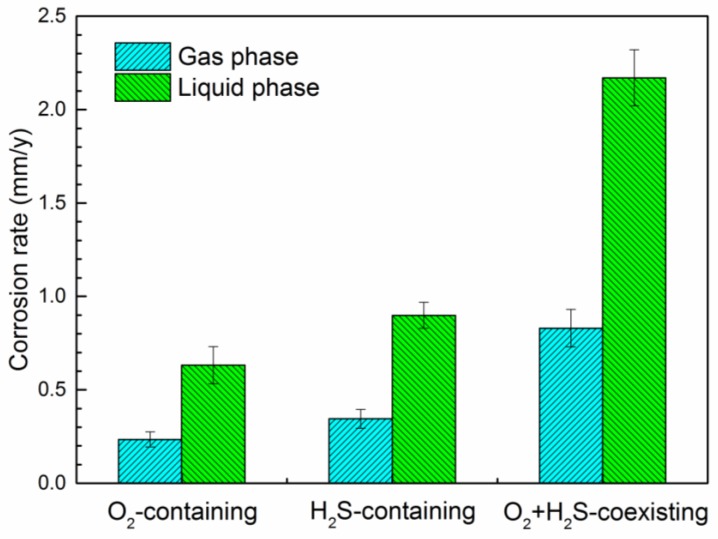
Corrosion rates of G20 steel in different conditions.

In gas phase, it is seen that the corrosion rate in H_2_S-containing environment is higher than that in O_2_-containing environment (Figure 3), i.e., in the presence of 0.21 MPa O_2_, the average corrosion rate in gas phase is 0.235 mm/y, while this value is as high as 0.345 mm/y in the presence of 600 ppmv H_2_S. The corrosion rate increases up to 0.83 mm/y when O_2_ and H_2_S co-exist in the environment. This indicates that the coexistence of O_2_ and H_2_S makes the environment more aggressive than the presence of single component, either O_2_ or H_2_S. This result is in good agreement with the findings of Sun et al. in water-saturated supercritical CO_2_ system [10]. They found that the accelerating effect of H_2_S on the corrosion of X65 steel was greater than that of O_2_, and the corrosion rates increased further with the coexistence of O_2_ and H_2_S. 

As a comparison, the corrosion rates of G20 steel in liquid phase under different conditions are also presented in Figure 3. Like in the gas phase, the corrosion rate of G20 steel in H_2_S-containing environment is higher than that in O_2_-containing environment, and the highest corrosion rate can be seen in O_2_-H_2_S-coexisting environment. Particularly, it is apparently that the corrosion rates in liquid phase are much higher than gas phase. For example, in the O_2_-H_2_S-coexisting environment, the corrosion rate of G20 steel is 2.17 mm/y in liquid phase, which is about four times higher than that in gas phase. Zhang et al. also found that the corrosion rate in liquid phase was always higher than that in gas phase in CO_2_-H_2_S-coexisting environment [19]. This is probably due to that the high conductivity of liquid leads to a higher electrochemical reaction rate than that in gas phase where only condensed water adsorbed on the surface of specimens.

### 3.2. Microstucture and Composition of Corrosion Products

#### 3.2.1. O_2_-Containing Environment

**Figure 4:**
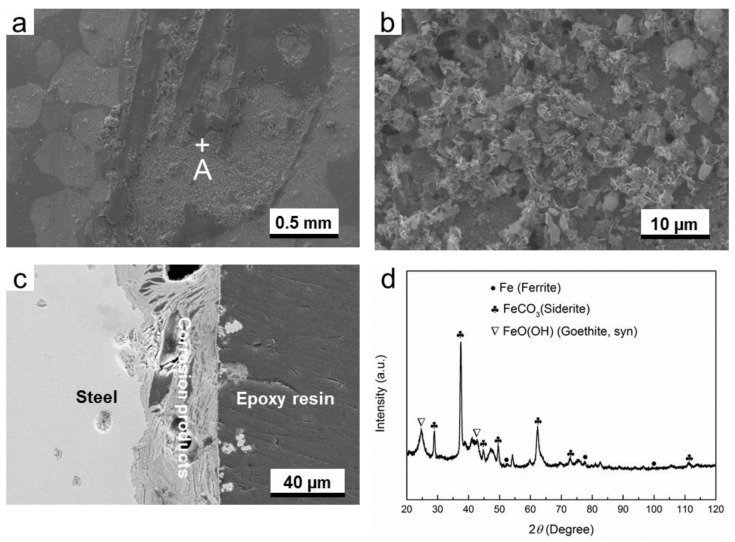
The characterization of corrosion products on G20 steel in gas phase in O_2_-containing environment: (**a**) SEM image of the surface of corrosion products; (**b**) the higher magnification of region A marked in (**a**); (**c**) corrosion-sectional image of corrosion product layer and (**d**) XRD pattern of the corrosion products.

As is shown in Figure 4a,b, the distribution of corrosion products is not homogenous, and some pores can be seen from the surface image of the corrosion products. Moreover, the pores and cracks can also be seen from the cross-sectional image of the corrosion product layer (Figure 4c), suggesting that this corrosion product layer is poor in corrosion protection. The XRD results identified that the corrosion products are mainly composed of FeCO_3_ (siderite) and FeO(OH) (goethite), as is shown in Figure 4d. The characterization peaks of ferrite are attributed to the substrate material. This result is in good agreement with the EBSD results (Figure 1), in which the ferrite is the dominant phase of G20 steel.

**Figure 5:**
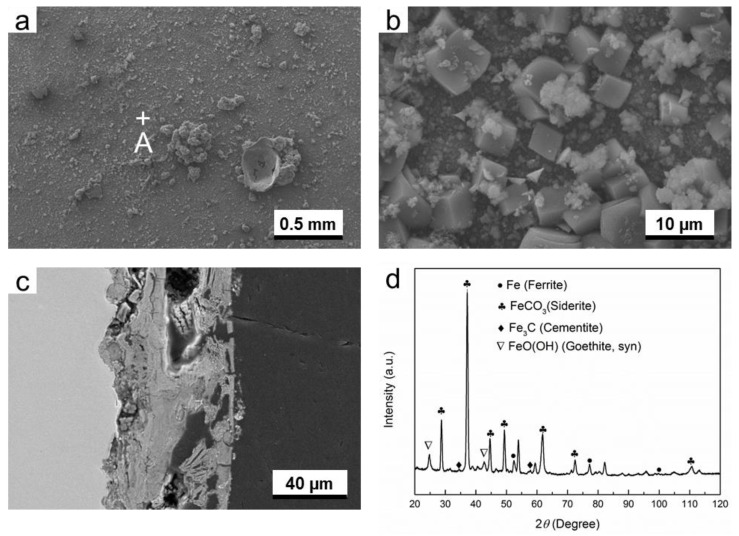
The characterization of corrosion products on G20 steel in liquid phase in O_2_-containing environment: (**a**) SEM image of the surface of corrosion products; (**b**) the higher magnification of region A marked in (**a**); (**c**) corrosion-sectional image of corrosion products layer, and (**d**) XRD pattern of the corrosion products.

In the corresponding liquid phase in O_2_-containing environment, it is found that the particles with different sizes are distributed on the surface of corrosion product layer (Figure 5). From the image with higher magnification as shown in Figure 5b, the prism-shaped crystals are distributed separately on the surface layer. The cross-sectional SEM image shows that lots of pores and cracks are presented in the corrosion product layer. This means that a high corrosion rate (0.632 mm/y) of G20 steel in liquid phase in O_2_-containing environment must be obtained, because the corrosion product layer could not supply enough protection for the substrate. The diffraction peaks of ferrite, FeCO_3_, Fe_3_C (cementite), and FeO(OH) can be seen in the XRD pattern shown in Figure 5d. Fe_3_C is the inclusions in the microstructure of the G20 steel, which has also been found in EBSD results (Figure 1). 

In O_2_-containing environment, the thickness of corrosion product layer formed in gas phase is smaller than liquid phase. However, the corrosion product layer in gas phase is less porous than liquid phase (see Figure 4c and Figure 5c). The difference in microstructure between both conditions probably causes the difference in corrosion rate. The XRD results indicate that composition of corrosion products for both gas and liquid phase is mainly composed of FeCO_3_ and FeO(OH). 

#### 3.2.2. H_2_S-Containing Environment

**Figure 6:**
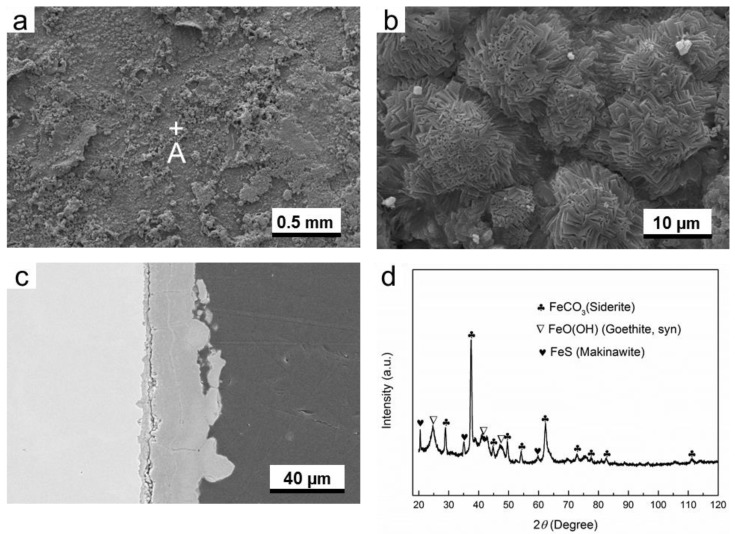
The characterization of corrosion products on G20 steel in gas phase in H_2_S-containing environment: (**a**) SEM image of the surface of corrosion products; (**b**) the higher magnification of region A marked in (**a**); (**c**) corrosion-sectional image of corrosion product layer, and (**d**) XRD pattern of the corrosion products.

It can be seen that the microstructure of corrosion product layer is different from that formed in O_2_-containing environment (Figure 6). There are a lot of needle-shaped substances distributed on the surface of corrosion products layer (Figure 6b). Numerous small cracks and pores can be seen, as shown in Figure 6c. Furthermore, the gap between the corrosion product layer and the substrate suggests poor adhesion between them. Therefore, the corrosive species can contact directly the substrate, and, subsequently, the corrosion occurs continuously. In the presence of H_2_S, the composition of corrosion product layer is more complicated, which consists of FeCO_3_, FeO(OH), and FeS (Makinawite). 

**Figure 7:**
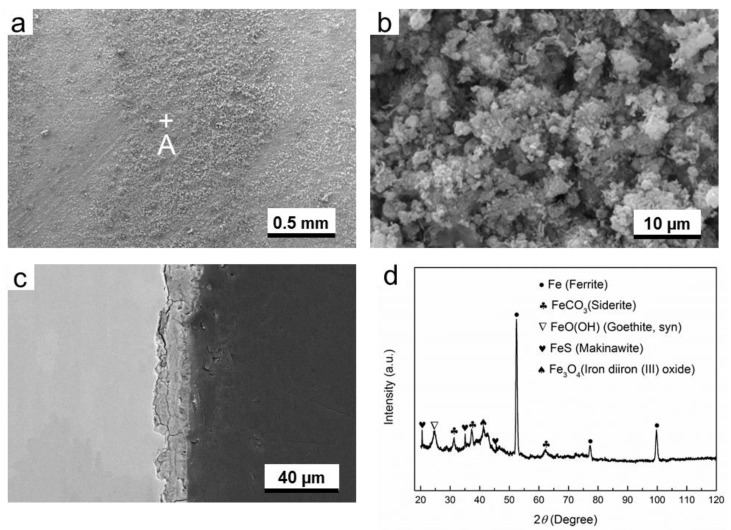
The characterization of corrosion products on G20 steel in liquid phase in H_2_S-containing environment: (**a**) SEM image of the surface of corrosion products, (**b**) the higher magnification of region A marked in (**a**), (**c**) corrosion-sectional image of corrosion product layer, and (**d**) XRD pattern of the corrosion products.

In the corresponding liquid phase, it can be seen that the structure of the corrosion products is loose, because large amount of pores and cracks are present, as shown in Figure 7b,c. The thickness of corrosion product layer is smaller than that formed in gas phase. This is probably due to the fact that the formed corrosion products are easily flaked off, and lots of corrosion products have fallen down into the solution. The presence of very strong diffraction peaks for ferrite in XRD pattern also confirms that the corrosion product layer is very thin, as is shown in Figure 7d. The composition of corrosion product formed in liquid phase is almost identical to that formed in gas phase, i.e., the corrosion products are composed of FeCO_3_, FeO(OH), and FeS. However, the Fe_3_O_4_ (iron oxide) is present occasionally in this condition. 

In H_2_S-containing environment, it can be easily understood that the formation of FeCO_3_ and FeS resulted from the presence of CO_2_ and H_2_S in the environment, respectively. According to the findings of Gao et al., the formation of Fe_3_O_4_ in H_2_S-containing environment at high temperatures is common [20], but in this work the temperature is only 60 °C. Therefore, the occasional presence of Fe_3_O_4_ may be attributed to the transformation from other iron compounds, such as the oxidation of FeS [21]. FeO(OH) is possibly transferred from the oxidation of Makinawite [21], because the contact between air and corrosion product cannot be completely avoided during the period from the end of experiment in autoclave to the time of XRD measurement.

#### 3.2.3. O_2_-H_2_S-Coexisting Environment

**Figure 8:**
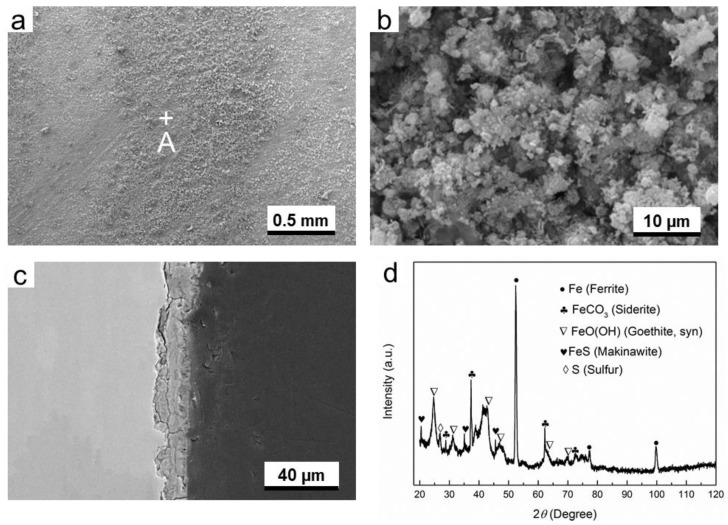
The characterization of corrosion products on G20 steel in gas phase in O_2_-H_2_S-coexisting environment: (**a**) SEM image of the surface of corrosion products, (**b**) the higher magnification of region A marked in (**a**), (**c**) corrosion-sectional image of corrosion product layer, and (**d**) XRD pattern of the corrosion products.

As shown in Figure 8a–c, it is obvious that numerous cracks are present in the corrosion products layer. At the same time, a remarkable gap between the corrosion product layer and the substrate can be seen (Figure 8c), meaning a poor adhesion between them. Therefore, it can be considered that the protection ability of the corrosion product layer is extremely low when O_2_ and H_2_S coexist in the environment. Therefore, the highest corrosion rate (0.83 mm/y) in gas phase was obtained in this condition (Figure 3). The XRD results (Figure 8d) demonstrate that the corrosion products are composed of FeCO_3_, FeO(OH), FeS, and S (element sulfur). 

**Figure 9:**
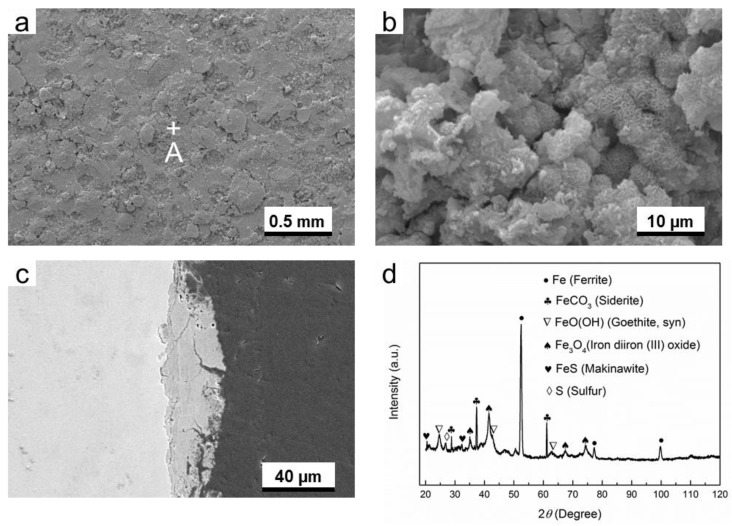
The characterization of corrosion products on G20 steel in liquid phase in O_2_-H_2_S-coexisting environment: (**a**) SEM image of the surface of corrosion products, (**b**) the higher magnification of region A marked in (**a**), (**c**) corrosion-sectional image of corrosion products layer, and (**d**) XRD pattern of the corrosion products.

Compared with the gas phase, the corrosion product layer in liquid phase is thinner, and its distribution is not homogenous (Figure 9). This is probably because the corrosion products are very loose, and they are easily flaked off from the substrate. Otherwise, the thickness of corrosion products should be the thickest, because in this case it shows the highest corrosion rate (2.17 mm/y). After the experiment in autoclave, large amount of corrosion products was found at the bottom of the autoclave. This phenomenon also supports our speculation. The composition of corrosion products formed in liquid phase is almost the same with the gas phase. However, small amount of Fe_3_O_4_ is also present in this condition.

As compared with the O_2_-containing environment and H_2_S-containing environment, the corrosion product layer formed in O_2_-H_2_S-coexisting environment is more porous, suggesting a poorer protection performance. The biggest difference in composition of corrosion products among them is that the presence of element sulfur. The element sulfur may be produced from the reaction between H_2_S and O_2_, or the reaction between H_2_S and Fe^3+^ [10], or the transformation of FeS. This will be discussed in the next section.

## 4. Discussion

### 4.1. Corrosion Reactions and the Formation Mechanisms of Corrosion Products in Flue Gas Injection Environment

The electrochemical reactions of steel in CO_2_-O_2_-H_2_S-coexisting environment are rather complicated, because there are various species in the solution, such as O_2_, H^+^, H_2_CO_3_, CO_3_^2^^−^, HCO_3_^−^, H_2_S, HS^−^, and S^2^^−^ [19,22,23,24,25]. These species can directly or indirectly participate in the corrosion reactions. Depending on the test conditions such as solution pH, and the partial pressure of corrosive gases, the following equations may be involved in the cathodic process:

O_2_ + 2H_2_O + 4e^−^ → 4OH^−^, (2)

2H^+^ + 2e^−^ → H_2_, (3)

2H_2_CO_3_ + 2e^−^ → H_2_ + 2HCO_3_^−^, (4)

2HCO_3_^−^ + 2e^−^ → H_2_ + 2CO_3_^2^^−^, (5)

2H_2_S + 2e^−^ → H_2_ + 2HS^−^, (6)

2HS^−^ + 2e^−^ → H_2_ + S^2^^−^. (7)

The anodic process is primarily the dissolution of iron:

Fe → Fe^2+^ + 2e^−^, (8)

In addition, the formation of corrosion product through the solid state reaction can also be considered as parts of anodic process [19]:

Fe + H_2_CO_3_ → FeCO_3_ + 2H^+^ +2e^−^, (9)

Fe + H_2_S → FeS_1__−x_ + xHS^−^ + (2 − x)H^+^ + 2e^−^. (10)

Figure 4, Figure 5, Figure 6, Figure 7, Figure 8 and Figure 9 show that corrosion products are present on G20 steel surface in each condition. Generally, the formation of corrosion products follows the solid state reaction mechanism and/or precipitation mechanism. The corrosion products formed in different conditions in this work include FeCO_3_, FeO(OH), FeS, Fe_3_O_4_, and S.

FeCO_3_ is a common CO_2_ corrosion product. Once Fe^2+^ and CO_3_^2^^−^ are present at the steel/solution interface at sufficiently high concentrations, which make the product of [Fe^2+^] × [CO_3_^2^^−^] exceed the solubility product of FeCO_3_, precipitation and crystal growth will occur [26], as described in Equation (11):

Fe^2+^ + CO_3_^2^^−^ → FeCO_3_. (11)

In this study, FeCO_3_ can be detected in the corrosion product layer formed in all the conditions. This is attributed to the relatively high CO_2_ partial pressure and high pH in the experimental environment. The CO_2_ partial pressure in each condition was up to 2.25 MPa, and the pH in corrosive solution was 7.0, which favored the formation of FeCO_3_ [26].

The formation of FeO(OH) resulted from the reactions related to O_2_. Yamashita et al. employed in situ XRD to detect the composition of corrosion products on iron in the O_2_-containing environment and found the initial corrosion products should be the mixture of Fe(OH)_2_ and Fe(OH)_3_, which are formed as the following equations [27]:

Fe^2+^ + 2OH^−^ → Fe(OH)_2_, (12)

4Fe(OH)_2_ + O_2_ + 2H_2_O → 4Fe(OH)_3_. (13)

These initial corrosion products are subsequently transformed to β-FeO(OH), α-FeO(OH), γ-FeO(OH), and/or Fe_3_O_4_, depending on the environment. In this study, α-FeO(OH) (goethite) was only detected (Figure 4, Figure 5, Figure 6, Figure 7, Figure 8 and Figure 9). It should be pointed out that small amount of FeO(OH) in the corrosion products formed in H_2_S-containing environment was also detected (Figure 6 and Figure 7). This is possibly due to the transfer from FeS (Makinawite) to FeO(OH) when the FeS is exposed to the O_2_-containing environment [21,28,29]. During the period from the end of experiment in autoclave to the time of XRD measurement, the contact between air and corrosion product cannot be completely avoided. Therefore, this speculation could be true.

Mackinawite (FeS) is widely considered to be the initial corrosion product in H_2_S corrosion due to its rapid formation kinetics [30,31]. It is generally considered that it can convert into other types of iron sulfide due to its meta-stability [31]; however, in this work only Mackinawite was detected in the corrosion product formed in H_2_S-containing environment and O_2_-H_2_S-coexisting environment, as is shown in Figure 6, Figure 7, Figure 8 and Figure 9. The formation of FeS can be expressed as the following equation:

Fe^2+^ + HS^−^ → FeS + H^+^. (14)

Like the formation of FeCO_3_, as long as Fe^2+^ and HS^−^ are present at the steel/solution interface at sufficiently high concentrations that make the value of [Fe^2+^] × [HS^−^]/[H^+^] exceed the solubility product of FeS, precipitation and crystal growth will occur. As mentioned above, FeS can be readily oxidized to form FeO(OH) when it is exposed to an O_2_-containing environment, as expressed in Equation (15) [30]:

4FeS + 3O_2_ + 2H_2_O → 4FeO(OH) + 4S. (15)

Therefore, the FeO(OH) in H_2_S-containing environment can be detected. Upon further exposure to O_2_-containing environment, the FeO(OH) can transform to Fe_3_O_4_ [30]. Smith et al. even proposed that FeS may be rapidly oxidized to Fe_3_O_4_ and sulfur by [32]:

FeS + 3O_2_ → Fe_3_O_4_ + S. (16)

That is why a small amount of Fe_3_O_4_ could be detected in H_2_S-containing environment (Figure 7 and Figure 9). 

In O_2_-H_2_S-coexisting environment, the formation of elemental sulfur probably resulted from the reactions between H_2_S and O_2_ and Fe^3+^ [10,33,34,35]:

2H_2_S + O_2_ → 2S + 2H_2_O, (17)

H_2_S + 2Fe^3+^ → S + 2H^+^ + 2Fe^2+^. (18)

At the same time, the transformation of FeS in O_2_-containing environment can also generate the elemental sulfur, as expressed in Equations (15) and (16).

### 4.2. The Coupling Effect of O_2_ and H_2_S on the Flue Gas Injection Corrosion

Compared with the O_2_-containing environment and H_2_S-containing environment, the weight loss results indicate that the coexistence of O_2_ and H_2_S causes a more obvious accelerating effect on corrosion rate of G20 in both gas phase and liquid phase. Taking the gas phase as an example, the corrosion rates are 0.235 mm/y and 0.345 mm/y in O_2_-containing environment and H_2_S-containing environment, respectively. However, the corrosion rate in O_2_-H_2_S-coexisting environment sharply increases up to 0.830 mm/y, which is much higher than the sum of corrosion rates in O_2_-containing environment and H_2_S-containing environment (Figure 3). This means that there is an interaction effect between O_2_ and H_2_S during the corrosion process, resulting in the more serious corrosion. Sun et al. thought that there was a synergistic effect between O_2_ and H_2_S on corrosion of steel in supercritical CO_2_ system [10]. In flue gas injection environment, a similar tendency of corrosion rate has been found in this work.

On the one hand, as expressed in Equations (2) and (6), O_2_ and H_2_S can directly participate in the cathodic reaction of steel, thereby increasing the corrosion rate. For H_2_S, following its dissolution in the solution, the pH will decrease and the corrosion rate will increase further. On the other hand, the reaction between O_2_ and H_2_S can generate elemental sulfur, which significantly accelerates the corrosion of carbon steel [10,34,35,36]. The corrosion mechanism of steel triggered by elemental sulfur has not yet reached consensus understanding. Different researchers have proposed different mechanisms, such as the direct reaction of elemental sulfur with iron [35], the electrochemical reaction of polysulfide with iron [36], and the corrosion caused by the formation of acid due to the hydrolysis of sulfur [37]. Regardless of the corrosion mechanism, elemental sulfur generated from the reaction of O_2_ and H_2_S can further accelerate the corrosion rate of steel. Therefore, a coupling effect on corrosion of G20 steel in flue gas injection environment has been found. The exact accelerating mechanism of elemental sulfur on corrosion still needs further investigation.

## 5. Conclusions

In simulating the flue gas injection environment, the coupling effect of O_2_ and H_2_S on corrosion of G20 steel has been found. In the gas phase environment, the corrosion rates in O_2_-containing environment and H_2_S-containing environment are 0.235 mm/y and 0.345 mm/y, respectively. However, the corrosion rate in the O_2_-H_2_S-coexisting environment is as high as 0.83 mm/y, which is much higher than the sum of corrosion rates of both the O_2_-containing environment and the H_2_S-containing environment. Similarly, the corrosion rate in liquid phase in the O_2_-H_2_S-coexisting environment is also higher than the sum of corrosion rate in both environments. It can be considered that this coupling effect resulted from the presence of elemental sulfur, which was mainly produced from the reaction between O_2_ and H_2_S.

The corrosion product of G20 formed in simulating flue gas injection environment consists of FeCO_3_, FeO(OH), FeS, Fe_3_O_4_, and S. It is also found that the corrosion product layer is porous and non-protective. Furthermore, the generation of elemental sulfur increases the corrosion of steel.

## Figures and Tables

**Table 1:** The ionic compositions of corrosive solution.

Ions	Concentration (mg/L)
Ca^2+^	125
Mg^2+^	35
Cl^−^	3940
HCO_3_^−^	1894
SO_4_^2−^	134
Na^+^ + K^+^	3126

**Table 2:** Test conditions of corrosion of G20 steel in simulating flue gas injection environment.

No.	Temperature (°C)	Pressure (MPa)	CO_2_ (MPa)	O_2_ (MPa)	H_2_S (ppmv)	Gas/Liquid Phase
1	60	15	2.25	0.21	0	Gas
2	60	15	2.25	0	600	Gas
3	60	15	2.25	0.21	600	Gas
4	60	15	2.25	0.21	0	Liquid
5	60	15	2.25	0	600	Liquid
6	60	15	2.25	0.21	600	Liquid
